# The Dental Hygienist in Orthodontic Practice*Read before the Alumni Society of the Dewey School of Orthodontia, April 1-3, 1920.

**Published:** 1920-12

**Authors:** H. B. Hamilton

**Affiliations:** Ithaca, New York


### THE DENTAL HYGIENIST IN ORTHODONTIC PRACTICE*

BY H. B. HAMILTON, D.D.S., ITHACA, NEW YORK

Discussing a paper on Oral Prophylaxis by Dr. Nelson at the St. Louis meeting of this Society the writer mentioned the use of the dental hygienist in his practice. A number of the members afterward made inquiries about the hygienist and evinced considerable interest in this aid to our work. The interest then shown is the excuse for this paper.

Ever since orthodontia became a specialty of dentistry there has been more or less controversy between the dentist and the orthodontist as to the effect on the teeth by the wearing of appliances. The dentist has blamed all sorts of injuries to both hard and soft tissues to the wearing of appliances. The orthodontist has claimed that proper appliances can cause no injury.

The orthodontic appliances in themselves, if properly designed, constructed and applied, and with the proper care, can cause no injury, but if any of these factors are at fault, there is undoubtedly danger to the oral tissues.

On the other hand, the orthodontist is constantly finding cavities and imperfections that the dentist overlooks. Unless these are cared for before the orthodontic work is begun, they may later be attributed to the appliance.

We also find the greatest difference in tooth structure and its liability to damage. In some mouths there seems to be almost a complete immunity to caries, while in others it will show evidence on the slightest provocation. There is also the greatest difference in the care which

* Read before the Alumni Society of the Dewey School of Orthodontia, April 1-3, 1920.

the patient gives to his teeth. Some patients are able to keep their teeth absolutely clean no matter how crude or complicated the appliance is, while others seem unable to keep them in a state that can be said to approach cleanliness, even without appliances. Instruction and continual lecturing will not entirely overcome this fault in all cases.

Before starting a case the mouth should be placed in the best of condition by the family dentist. With the placing of appliances the responsibility for the mouth rests largely on the orthodontist. Prophylactic treatment seems to be the best method of meeting this responsibility. To do this work oneself becomes burdensome, and as the great majority of our patients are school children, it is not always possible to give every patient the prophylactic attention that may be needed, and make necessary adjustments in the appliances for all the patients that have to be cared for out of school hours. It is here that the dental hygienist is of the greatest value, not only in relieving us of this burdensome task, but what is more important in conserving our time.

A dental hygienist is a person licensed by the state to remove stains and accretions from the exposed surfaces of the teeth under the supervision of a practitioner of dentistry, and to teach oral hygiene. The chief effort of the hygienist is to make the teeth clean and to instruct the patient so that he will maintain them in a cleanly condition. It is not necessary to go into the technic, but sufficient to say that the hygienist will do this work more thoroughly and efficiently than the average dentist.

Neither is it necessary to go into the subject of the training of the hygienist as the movement is now so well known in the dental profession. It may be said that the course is certainly thorough and that the student gets a large amount of practical experience. The various institutions teaching dental hygienists have orthodontic clinics and it should be and probably is easy for the student to get some actual experience with orthodontic practice. The following quotations from letters from the heads of some of these institutions show that they are alive to the possibilities:

"We give them talks on the need and value of their services to the orthodontist."

"The dental hygienist receives theoretical and practical training in the handling of children, the importance of child hygiene and the effect of any oral abnormality upon the health of the child."

"The dental hygienist receives intensive training in oral prophylaxis."

"She is also instructed as to how to teach children to keep their mouths clean and can give them wash bowl demonstrations in the office."

"She receives lectures in dental anatomy, and in the tooth morphology class must carve a complete set of teeth by hand."

"Lectures on occlusion and malocclusion are given."

"She is given instruction in making and separating models, cleaning appliances and in thorough prophylaxis of the mouths of orthodontic cases."

That this instruction is bearing fruit is proved by the fact that I have had several cases referred to me by our local school hygienist when the family dentist had been negligent or overlooked the malocclusion.

In addition to instruction noted in the above quotations, the hygienist is taught to make an examination of the mouth, to note any defects and abnormalities, and to properly chart such deficiencies. She also receives instruction in X-ray work: something of the machine, the technic of taking the picture, the developing and printing.

In our practice after the preliminary arrangements are made the child is turned over to the hygienist to prepare for the taking of impressions. She takes the child and seats him in the chair and while explaining the procedure endeavors to interest him and gain his confidence.

The orthodontist may be able to do this just as successfully as the hygienist, but the latter is oftentimes able to accomplish this quicker and more effectively because a child will usually respond quicker to the efforts of a woman.

Before the bands are fitted and cemented the hygienist can remove the separating wires and thoroughly cleanse the teeth to be banded and the adjoining teeth. Before the appliances are put on she should thoroughly cleanse all of the teeth. After the appliances are in place she should teach the child how to care for the teeth with the appliances on and see that proper care is taken. If proper care is not taken it is her duty to persist in the effort with the child until a reasonable result is obtained.

While the case is in progress and comes in for adjustment it is examined and if necessary to remove the appliance the case may be turned over to the hygienist for the removal and to give the proper prophylactic treatment. During this treatment she should note carefuly the condition of the cemented bands, the evidence of beginning cavities, an irritated gum, and if there is anything suspicious to immediately call the attention of the operator to it.

It is not intended that the operator should rely entirely on the hygienist for this, but should in fact look for these conditions at each visit, but the prophylactic work of the hygienist should include all this. Thus she may note something that might be overlooked by the operator as it is of course more easy to observe these things when giving attention to each individual tooth.

While the hygienist is at work on the first case, the operator takes up the second one and gets that under way. By this time the hygienist is through with the first case and the operator returns and replaces the appliance with such adjustments as are necessary. And so on with the other cases.

I do not mean to say that every child receives a prophylactic treatment at every visit. The frequency of the treatment depends on a number of factors, such as the amount and nature of the appliance, the care the patient gives to the mouth, the diet of the patient, etc. It is desirable, however, that at every visit the mouth should be looked over and if there is any evidence of neglect at any point it should be corrected and the child's attention called to it. By this constant vigilance possibly more prophylactic work in the aggregate may be done than is absolutely necessary, yet the individual treatments may require only a few minutes and the final results are most desirable. I am sure of this because I have had cases carried over a number of years with very little reparative work done during the period of treatment, while previously the dentist complained that it seemed almost impossible to keep the teeth in repair.

While the foregoing is the most important aid to the orthodontist, there is a great deal of time when patients are not available. Yet between times there are a thousand and one things to be done. Impressions are to be prepared, models to run and separate, and also to carve. The hygienist can easily be taught to do them, and if she has any mechanical skill can also be utilized to a more or less extent in the making of appliances.

There are also the books and records as well as the correspondence that may be turned over to the hygienist.

It is unnecessary to refer to the influence of the hygienist in the office and the many little ways in which she can keep things up to a higher standard. Being an operator she may appreciate these things a little better than the average assistant.

I do not know that I can close this paper better than by quoting Dr. Crosby, of New London: "The reason why we could not do without a hygienist is that we are in duty bound to bring the teeth through the period of orthodontic treatment in as good condition as we received them."—*Journal of Orthodontia and Oral Surgery.*

